# Influence of ultrasonic combined supercritical-CO_2_ electrodeposition process on copper film fabrication: Electrochemical evaluation

**DOI:** 10.1016/j.ultsonch.2021.105555

**Published:** 2021-04-20

**Authors:** Sabarison Pandiyarajan, Po-Ju Hsiao, Ai-Ho Liao, Muthusankar Ganesan, Shobana Sebstin Mary Manickaraj, Chen-Ta Lee, Sheng-Tung Huang, Ho-Chiao Chuang

**Affiliations:** a Department of Chemical Engineering and Biotechnology, National Taipei University of Technology, Taipei 10608, Taiwan; b Department of Mechanical Engineering, National Taipei University of Technology, Taipei 10608, Taiwan; c Graduate Institute of Biomedical Engineering, National Taiwan University of Science and Technology, Taipei, Taiwan; d Department of Biomedical Engineering, National Defense Medical Center, Taipei, Taiwan; e Department of Industrial Chemistry, Alagappa University, Karaikudi 630001, Tamil Nadu, India; f Ya De Li Technology Co., Ltd., Taipei 104031, Taiwan

**Keywords:** Ultrasonication, Electrodeposition, Surface studies, Electrocatalytic studies, Corrosion analysis, Supercritical-CO_2_

## Abstract

Introducing ultrasound irradiation to the electrodeposition process can significantly improve the physical and chemical properties of deposited films. Meanwhile, the beneficial effects from supercritical-CO_2,_ such as high diffusivity, high permeability, low surface tension, etc., would improve the electrodeposition process with better surface quality. In the shed of the light, the present work deals with the preparation of copper (Cu) films using the integrated techniques, i.e., ultrasonic-assisted supercritical-CO_2_ (US-SC-CO_2_) electrodeposition approach. For comparison, Cu films were also prepared by normal supercritical-CO_2_ (SC-CO_2_) and conventional electrodeposition methods. To investigate the characteristics of Cu films, surface morphology analysis, roughness analysis, X-ray diffraction studies (XRD), Linear polarization, electrochemical impedance spectroscopy (EIS), and cyclic voltammetry (CV) were performed. In this work, EIS analysis was utilized for interfacial charge transfer resistance analysis with 5 mM [Fe(CN)_6_]^−3/−4^ redox system and corrosion analysis with 3.5 wt% NaCl solution. The observed results revealed that the film prepared with the US-SC-CO_2_ method have superior properties than those produced by normal SC-CO_2_ and conventional methods. Due to the combination of US-SC-CO_2_, the cavitation implosion occurs rapidly that enriches the deposited film quality, such as sufficient grain size, smoother surface, enhanced corrosion resistance, and charge carrier dynamics. On the other hand, the ultrasound effect with SC-CO_2_ helped to remove the weakly adhered metal ions on the electrode’s surface.

## Graphical abstract

**Figure:**
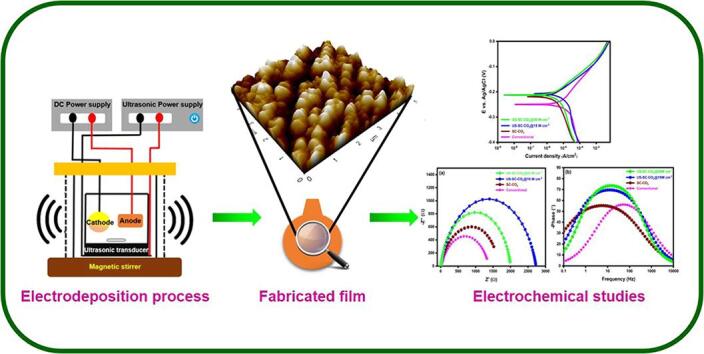

## 1 Introduction

Electronic device usage has been rapidly increased and evolves concerning compact size, weightless, high-ampacity, and high reliability in recent years. In this concern, surface deposition or coating has been employed to improve the performance and increase the material’s life span. Among the several techniques, electrodeposition has good exposure to produce a thin layer surface coating with low-cost, flexible, and economical usage [1], [2], [3], [4]. Moreover, it plays a significant role in the industrial sector to cast-off semiconductor and micro-electromechanical systems (MEMS) [5]. Due to the excellent electrical conductivity, copper (Cu) is a historically popular material with many engineering applications. The properties of the Cu film can be tuned by the electrodeposition parameters such as current density, electrolyte bath components, temperature, etc. [6], [7], [8], [9]. Although, hydrogen adsorption is inevitable in the conventional process, which leads to insufficient surface property and causes failure in electronic products. To address the issue, surfactants and additives are usually added to the electrolyte bath to improve the coating properties. However, they could affect the purity of coatings and very challenging to recycle the electrolyte after the electrodeposition process.

Supercritical fluid (SCF)-based electrodeposition process is considered the best replacement for crystal modifier additives and surfactants. The supercritical fluid research has been receiving extensive research interest since it was first noticed in 1822 [10]. When the SCF exists above its critical point, it provides several advantages during the deposition process, such as high diffusivity, high density, low surface tension, and low viscosity. Among the various SCFs, the most popular choice is carbon dioxide (CO_2_) because of the low critical point, abundant availability from the industrial by-product, and non-toxicity compared to other substances [11]. Introducing the supercritical-CO_2_ into the electrolyte bath can significantly improve the electrodeposition through a microbubble explosion. It helps to accelerate the mass transfer of metal ions to the cathode [12]. Moreover, it is a good substitute for organic solvents. In 2003, Sone et al. introduced the supercritical carbon dioxide (SC-CO_2_) to the electroplating process and achieved a uniform surface, adequate grain size, and higher hardness of the nickel coating [13]. They have recently reported that the changes in pressure conditions can influence grain structure of Cu films and found that the periodic plating characteristic (PPC) exists in the SC-CO_2_ electroplating process [14], [15], [16], [17], [18], [19], [20]. Similarly, Li et al. also used an emulsified SC-CO_2_ bath for the high aspect ratio micro-hole filling in Ni-P alloy [21]. However, the solubility of hydrogen in SC-CO_2_ is much higher than that in aqueous solution, which lowers the electrical conductivity of SC-CO_2_ and leads to limiting the deposition rate with limiting current.

In recent decades, ultrasonic-assisted electrodeposition has been widely used to improve the quality of electrodeposited materials [22], [23], [24]. It is due to the fact of high energy from the ultrasonic irradiation that creates cavitation effects such as mass transport processes, acoustic streaming, surface activation, and micro jetting formation [25]. When ultrasonic agitation is applied to the electrochemical cell, the primary acoustic cavitation effect occurs in the electrolyte medium, influencing the crystal growth and removing the hydrogen adsorption [26], [27]. Besides, the explosion of cavitation bubbles nearby the cathode surface directly affects nucleation mechanisms and provides a high density of nucleation sites, which increases the random nucleation growth [28], [29], [30]. In 2015, Tudela et al. investigated ultrasonic power's effect during the ultrasound-assisted electroplating of nickel coating. They found that higher power is not proportional to the highest impact. At the high power ultrasonication condition, the cavitation burst creates microjet formation, and the behavior of shock waves inside the electrolyte could affect the crystal growth process on the cathode surface, leading to variations in the coating characteristics [31]. Camargo et al. applied different combinations of current density and ultrasonic power to the electrodeposition of Zn-TiO_2_ film; the coating hardness was enhanced at a current density of 20 A/dm^2^ with ultrasonic power of 28 and 53 mW/cm^3^ [32]. Ataie et al. studied the effect of ultrasonic agitation on the composite coating and Al_2_O_3_ fraction properties. They found the reduced grain sizes due to the increase in ultrasonic power, enhancing the roughness, and wear resistance [33]. Although, the excessive ultrasonic irradiation could bring increased microjet effects, which damages the deposited coating surface up to the point of creating pinholes.

The combined techniques of ultrasonic irradiation and SC-CO_2_ condition will sum up the advantages mentioned earlier. They also influence surface morphologies, grain refinement, lower surface roughness, higher coating hardness, etc. The synergetic effect from ultrasonic agitation aided the supercritical carbon-dioxide (US-SC-CO_2_) process succeeding a more effectual emulsification effect and an attenuated cavitation behavior called “soft cavitation” [34], [35], [36], [37], [38], [39], [40]. Thus, in the present work, we have performed a detailed study of two different ultrasonic powers’ (15 and 30 W cm^−2^) under the SC-CO_2_ condition and the influence on the Cu film’s electrodeposition process. Furthermore, we also demonstrated that the electrochemical performance of coatings produced by ultrasonic agitation aided supercritical carbon-dioxide (US-SC-CO_2_) conditions with different power densities. Based on the results, it is anticipated that the ultrasonic technology can contribute to developing the electrodeposition process with eco-friendlier, enhancing physical and chemical properties.

## 2 Experimental methods

### 2.1 Materials

**Fig. 1:**
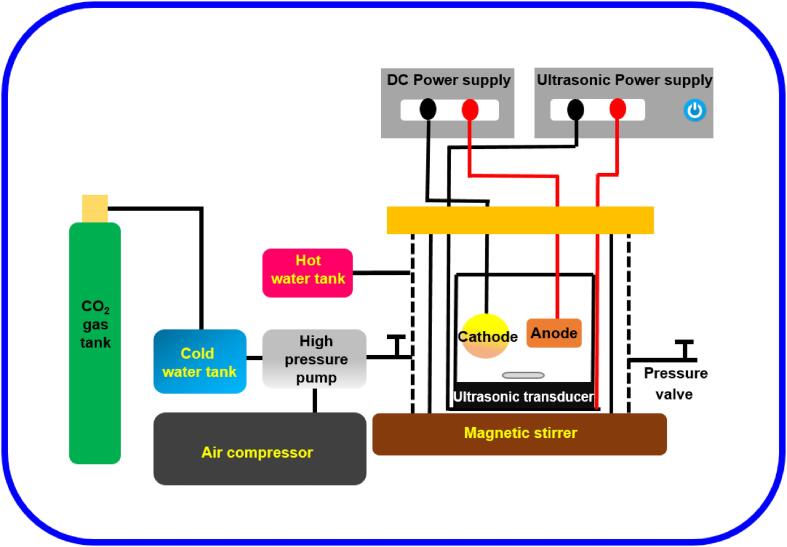
Schematic representation of ultrasonic modified high-pressure electrodeposition instrument setup.

A high-pressure machine was employed for the SC-CO_2_ and US-SC-CO_2_ electrodeposition process in this study, as shown in Fig. 1. For a US-SC-CO_2_ electrodeposition process, the reaction cell was mounted with a circular piezoelectric transducer to produce the ultrasonic waves with a frequency of 42 kHz. The electrolyte was prepared with CuSO_4_·5H_2_O (160 g/L) and H_2_SO_4_ (29.6 g/L). Here CuSO_4_·5H_2_O acts as a metal precursor, and H_2_SO_4_ acts as a stabilizing agent. The rectangular piece of Cu with a 20 × 25 × 5 mm dimension was used as an anode, and the circular brass with a diameter of 17 mm was taken as a cathode substrate. The distance between the cathode and anode is 10 mm. Prior to the electroplating, the substrates were ground with SiC paper of 800, 1500, and 2000 grit, then polished with alumina powder (1–0.03 µm particle sizes) until a mirror-like finish. Later, the samples were cleaned in a sonication bath with acetone and isopropyl alcohol for 5 min. Finally, the substrates were ready for the deposition process. The precise electrodeposition operating conditions are stated in Table 1. For the conventional electrodeposition, the same parameters were adopted, as mentioned in Table 1, with the exception of pressure and ultrasonic agitation.

**Table 1:** Cu electrodeposition parameters.

Parameter	Setting
Reaction volume	100 mL
Applied current density	3 A/dm^2^
Temperature	50 °C
Magnetic agitation	300 rpm
Deposition time	30 min
Critical pressure	2000 psi
Ultrasonic frequency	42 kHz (constant)
Ultrasonic power	15 and 30 W cm^−2^

### 2.2 Characterization of deposited Cu films

The deposited films’ surface morphology and structure were analyzed using a field emission scanning electron microscope (FE-SEM) (Sigma Essential by Zeiss). The crystallographic structure, grain size, and surface roughness were characterized using an X-ray diffractometer (XRD) (EMPYREAN by PANalytical) and an atomic force microscope (AFM, XE-100 by Park), respectively. The average grain size of the films was calculated by the Scherrer equation, as shown in Eq. (1),

(1) $$D=\frac{k\lambda }{\beta cos\theta }$$

where D is the average grain size, k is the shape factor (0.9 for dimensionless shapes), λ is the incident light wave generated by a Cu-Kα source known to be 0.15418 nm, θ is the diffraction angle, and β is the full-width half-maximum (FWHM), θ and β were measured from main diffraction peak. All the electrochemical studies were carried out in the Auto-Lab PGSTAT302N model by Metrohm with a three-electrode system. The electrodeposited Cu film was served as a working electrode, a platinum wire as a counter electrode, and Ag/AgCl as a reference electrode. The electrochemical impedance spectroscopy (EIS) and cyclic voltammetry (CV) were performed to evaluate charge transfer dynamics and the active surface area in a typical redox probe. Moreover, EIS and linear polarization scanning (LPS) were performed to study the corrosion performance in 3.5 wt% of NaCl electrolyte. EIS was conducted in the frequency ranging from 0.1 to 10 kHz, and LPS has scanned from OCP value of ±200 mV and 1 mV/s.

## 3 Results and discussion

### 3.1 Surface morphology studies

**Fig. 2:**
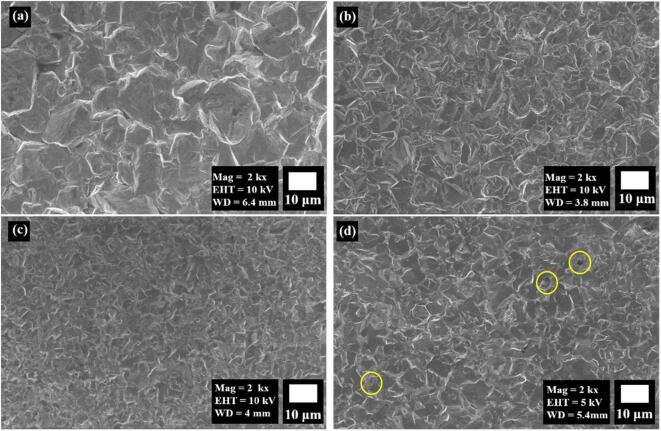
Surface morphology of (a) conventional, (b) SC-CO_2_, (c) US-SC-CO_2_ @ 15 W cm^−2^, (d) US-SC-CO_2_@ 30 W cm^−2^.

The film’s morphology produced with the conventional method shows a nodule-like structure with large grain size (Fig. 2a). In contrast, the Cu films deposited from the SC-CO_2_ and US-SC-CO_2_ method exhibited a compact coating surface with a smaller grain size than the conventional method, which is shown in Fig. 2(b–d). The Cu film’s smoother surface formed from the SC-CO_2_ condition is due to the suspended CO_2_ in the electrolyte replenishing the metal ions through microbubble explosion. Consequently, the flowability results in the size reduction in deposited grains and hinders theirs over deposition. Moreover, it predominantly reduces the H_2_ adsorption on the substrate and facilitates a compact arrangement [13], [16]. The US-SC-CO_2_ electroplating process shows a more delicate surface with a smaller grain size than SC-CO_2_ and conventional methods. With the introduction of ultrasonic agitation (15 W cm^−2^) to the SC-CO_2_ condition, the physical nature of the cavitation effect turns into soft cavitation behavior that enhances the mass transfer of metal ions from the electrolyte to the electrode by the diffusion-controlled reactions. However, the increased ultrasonic power (30 W cm^−2^) creates more energy from the cavitation effects, which affects the smoothness of the deposited films and also leads to surface roughness and pinholes formation (Fig. 2d) [40]. As a result, it can be concluded that the US power of 15 W cm^−2^ could be the optimum value for the better electrochemical deposition of the Cu film.

### 3.2 Surface roughness analysis

**Fig. 3:**
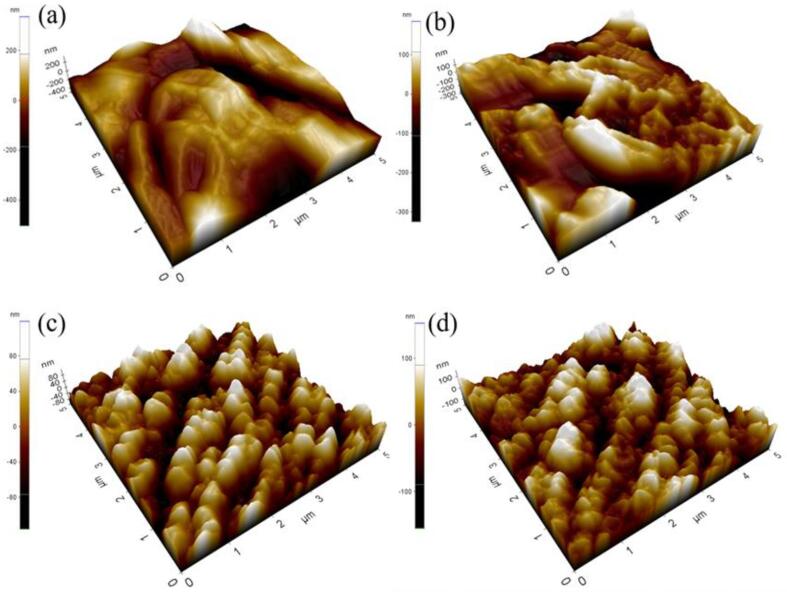
AFM images of films prepared by (a) conventional, (b) SC-CO_2_, (c) US-SC-CO_2_ @ 15 W cm^−2^, (d) US-SC-CO_2_@ 30 W cm^−2^.

**Fig. 4:**
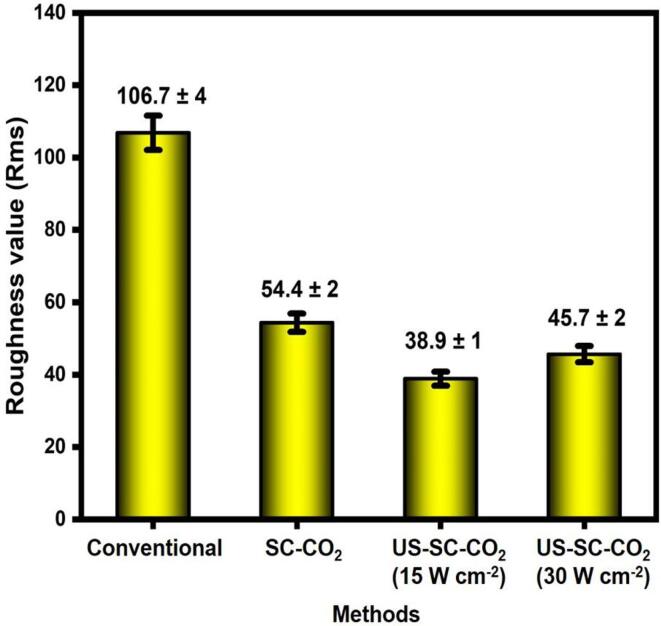
Roughness value of copper coatings obtained AFM images.

The AFM analysis was performed to observe the roughness details of Cu films in the 3D profile, as shown in Fig. 3. The 3D height differences seem larger on the surface, which corresponds to the conventional method (Fig. 3a). During the electrodeposition reaction, metal ions are deposited on the surface in a random manner, which leads to the aggregation of crystals with a large hump. In the SC-CO_2_ condition (Fig. 3b), the aggregation of the molecules was reduced by exploited microbubbles [16] and form a compact surface with lower roughness than the conventional method. Moreover, Fig. 3c displays the lower surface roughness than all other methods. It is mainly due to the influence of ultrasonic irradiation. Introducing the ultrasonic agitation generates an enhanced cavitation effect that results in lower surface roughness. Despite the advantages of the ultrasonic process, higher power does not provide smoother surface roughness. The main reason is that the higher ultrasonic power of 30 W cm^−2^ generates violent cavitation implosions, resulting in several pinholes and gap structures over the electrodeposited surface, compared with the 15 W cm^−2^ arrangement. The average surface roughness values measurements are illustrated as bar graphs in Fig. 4.

### 3.3 XRD analysis

**Fig. 5:**
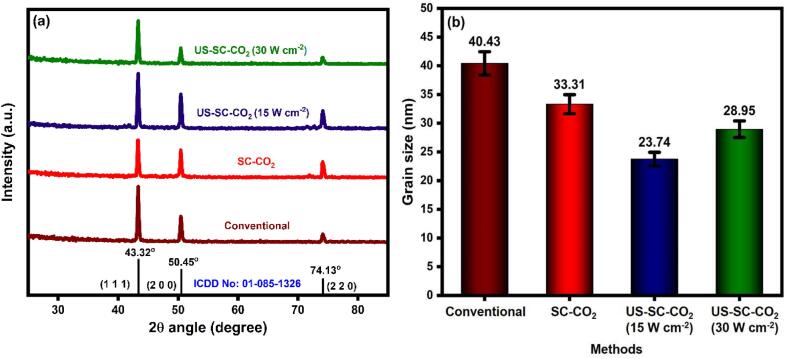
(a) XRD pattern of electrodeposited Cu films, (b) bar graph of calculated grain size along with error bar.

The observed XRD profile of all electrodeposited films is shown in Fig. 5(a). All the prepared Cu films have a preferred orientation of (1 1 1), (2 0 0), and (2 2 0) at 2θ angle of 43.32°, 50.45°, and 74.13°, which are well-matched with the standard pattern (ICDD No.: 01-085-1326) of pure Cu with the cubic crystal system. However, the Cu films’ peak intensity and width, prepared from the three different methods, are slightly different from each other, indicating the variations in crystallite size. Compared to the conventional method, SC-CO_2_ and US-SC-CO_2_ methods exhibited a slight decrease in peak intensity. This is due to the effect of microbubbles explosion from the supercritical CO_2_ affects nucleation growth during the electrodeposition. Notably, the observed diffraction pattern in US-SC-CO_2_-15 W cm^−2^ displays the most significant full width at half maximum (FWHM), which indicated that ultrasonic agitation would lead to the smallest grain size [16], [23], [24]. Similar to the previous reports, the grain sizes are calculated from the Scherrer equation [38], [39], [40], [41], [42]. The calculated average grain size values are presented in Fig. 5(b). Ultrasonic agitation hindered the poorly adhered metal ions and increased the more propagation of nucleation sites, resulting in grain refinement. While increasing the ultrasonic power, the microbubbles explosion gets faster due to the explosion of power. Meanwhile, increasing the ultrasonic power will make the thin diffusion layer on the cathode surface [43], [44]. The metal ion’s activity will be enhanced and reduced the concentration polarization, resulting in a slightly coarser surface finish. Thus, the US-SC-CO_2_ method with 15 W cm^−2^ power gained a smaller grain size of 23.74 nm, which could be expected to improve electrochemical studies’ performance.

### 3.4 Electrocatalytic studies

**Fig. 6:**
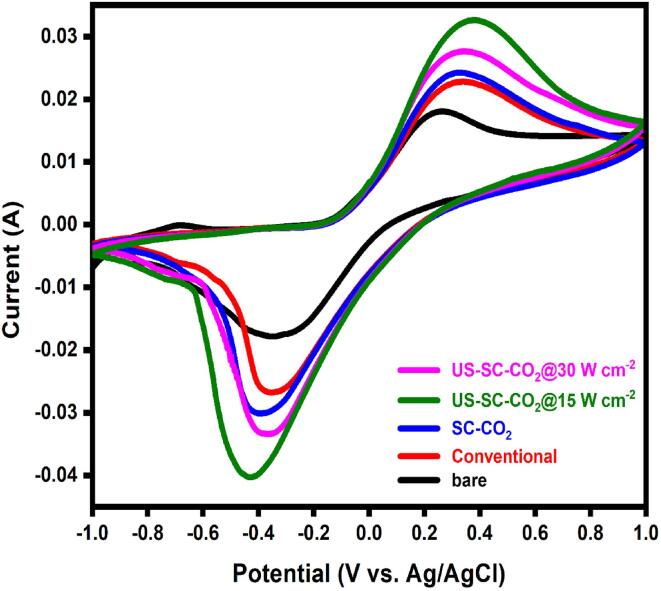
CV plot of fabricated films in the 0.1 M KCl solution containing 0.05 M [Fe(CN)_6_]^3−/4−^ redox probe at the scan rate of 100 mV s^−1^.

To explore the electron transmission features of the fabricated films, CV and EIS techniques were conducted. Both analyses were examined in a typical redox probe solution of 0.1 M KCl with 0.05 M [Fe(CN)_6_]^3−/4−^ at the scan rate of 100 mV s^−1^. The CVs of the deposited Cu films are shown in Fig. 6. Compared to the bare substrate, the Cu film from the conventional electrodeposition method exhibited a higher redox peak current response.

The result suggests that the electrodeposited Cu over the brass substrate significantly increases the electrocatalytic activity. Compared to the conventional method, the increased current response is observed at the Cu film obtained from the SC-CO_2_ process. In SC-CO_2_, the formed microbubbles accelerate the metal ions’ through the PPC effect that enhances the localized particulate flow near the cathode surface. As a result, grain refinement with more active site surfaces is generated, which increases the surface-to-volume ratio, thereby enhances the active surface area of the film [45].

Among the fabrication methods, the Cu film from the US-SC-CO_2_ (15 W cm^−2^) process exhibited a higher current response than SC-CO_2_ and conventional electrodeposition methods. The observed result confirms that ultrasonic power utilization enhances the electrocatalytic activity due to reducing the diffusion layer’s thickness and raising mass-transport of electroactive substances to the electrode surface. The combination of ultrasonic agitation with SC-CO_2_ further accelerates the metal ions in the electrolyte through the soft cavitation behavior, resulting in a homogenous distribution of ions and increased active site species on the grain boundary [38], [39]. In contrast, a further increase in the ultrasonic power (30 W cm^−2^) reduces the Cu film’s electrocatalytic performance. It might be due to the violent cavitation implosions at high ultrasonic power. Thus, it results in a rough surface and several pinholes over the electrodeposited surface, compared with the 15 W cm^−2^ arrangement. Further, it is evident that the electrocatalytic performance depends on their surface morphology. The observed performance is well consistent with the results obtained from the prepared Cu films’ FE-SEM images. The obtained results show the prominent role of the US-SC-CO_2_ electrodeposition method and its conditions for the efficient preparation of the Cu films.

**Fig. 7:**
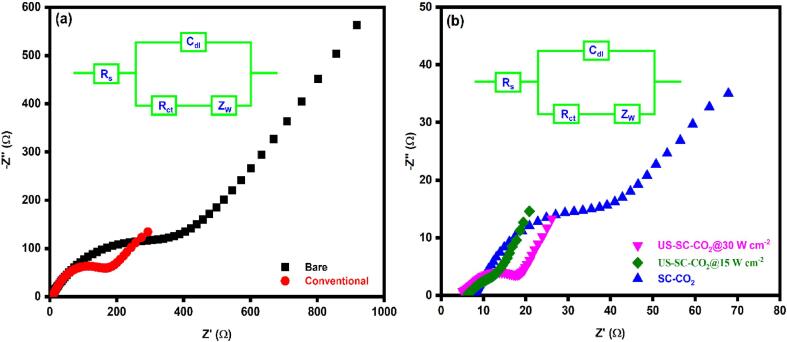
Nyquist plot of fabricated films in the 0.1 M KCl solution containing 0.05 M [Fe(CN)_6_]^3−/4−^ redox probe.

The electron transfer rate at the electrode/electrolyte interface was studied using EIS, and its results are shown in Fig. 7. The semicircle and a straight line describe the interfacial charge transfer resistance and Warburg diffusion layer, respectively. The obtained data is adopted to Randel’s equivalent circuit, as shown in the inset of Fig. 7a. The R_s_, R_ct_, C_dl_, and W stand for solution resistance, charge transfer resistance, double-layer capacitance, and Warburg impedance. The calculated R_ct_ values of fabricated films are presented in Table 2. In comparison, the film produced with US-SC-CO_2_ (15 W cm^−2^) possesses a low R_ct_ value, which implies higher electron transmission capability than others Fig. 7b. It is noteworthy that the increased ultrasonic power significantly increased the microjet effect, which affects the crystal growth process during electrodeposition. Thus, the smaller grain size possesses higher electron transmission efficiency; this resembles morphological studies. Here, we notice that the increase of ultrasonic power to 30 W cm^−2^ decreases the electrodeposited Cu film’s catalytic performance. The EIS results are well correlated with the corresponding CV results.

**Table 2:** Charge transfer resistance of fabricated films.

Electrodeposition Methods	R_ct_ (Ω)	Estimated error (%)
Bare substrate	633.06	3.72
Conventional	220.21	4.63
SC-CO_2_	53.77	5.31
US-SC-CO_2_ @ 15 W cm^−2^	12.54	2.26
US-SC-CO_2_ @ 30 W cm^−2^	18.86	6.19

### 3.5 Corrosion analysis

**Fig. 8:**
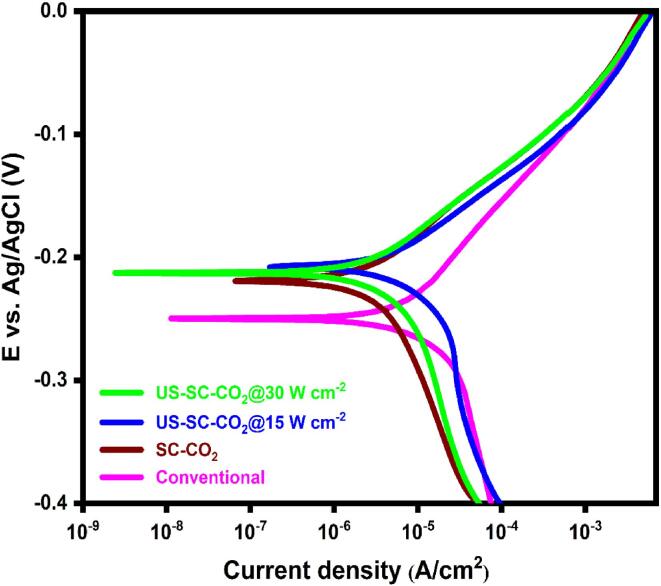
Tafel graph of fabricated Cu films.

Corrosion analysis is studied with linear polarization techniques and electrochemical impedance spectroscopy techniques with a 3.5 wt% of NaCl solution. Fig. 8 shows the Tafel extrapolation plot of all fabricated films. From the Tafel extrapolation, the corrosion potential (E_corr_) and current (I_corr_) were calculated. The observed E_corr_ and I_corr_ values of the electrodeposited Cu films from conventional, SC-CO_2,_ US-SC-CO_2_ (42 kHz @ 15 W cm^−2^) US-SC-CO_2_ (42 kHz @ 30 W cm^−2^) are −0.249 V/16.301 μA/cm^2^, −0.218 V/5.136 μA/cm^2^, −0.207 V/3.844 μA/cm^2^ and −0.212 V/4.845 μA/cm^2^ respectively. Based on the literature [38], [39], [40], the best corrosion resistance could be recognized by a lower corrosion current with lower potential (more positive region). According to the strategy, the best corrosion resistance was achieved by US-SC-CO_2_ (42 kHz @ 15 W cm^−2^) than other electrodeposition methods.

**Fig. 9:**
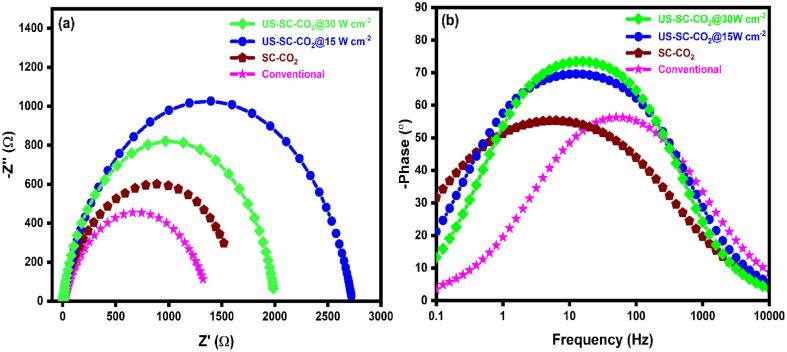
(a) Nyquist plot, (b) Bode plot of fabricated films.

**Fig. 10:**
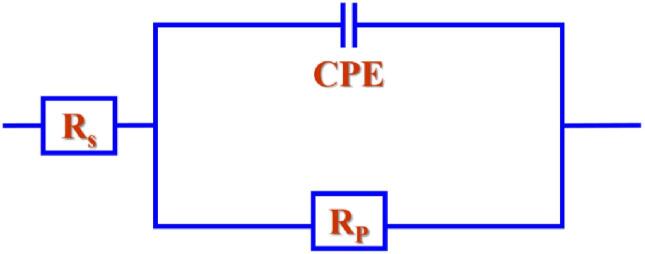
Electrical equivalent circuit model.

Fig. 9 shows the Nyquist and Bode plot of the fabricated film by conventional, SC-CO_2,_ and US-SC-CO_2_ methods. The EIS analysis data is adopted to the electrical equivalent circuit model (EEC) shown in Fig. 10. All the Nyquist plots and Bode plots appear with perfect capacitive loop and hump-like shape, which describes information about the polarization resistance. The electrical circuit elements such as solution resistance, polarization resistance, and constant phase elements are expressed as R_s_, R_p,_ and CPE. Further, the fitted results are presented in Table 3. The best corrosion resistance could be recognized by a large semicircle of the Nyquist plot and broad-spectrum with peak shifted to a lower frequency of Bode plot [46], [47]. In that fact, Cu film from the US-SC-CO_2_ (42 kHz @ 15 W cm^−2^) method shows a colossal semicircle and a broad spectrum of Nyquist and Bode plots and exhibiting a higher corrosion resistance performance than other prepared Cu films. Usually, the corrosion occurs through micro-pores and the high roughness surface of the film. When submerging the fabricated film into the 3.5 wt% NaCl solution, the chloride ions in the corrosive solution react with the Cu film. It produces Cu chloride side products, damaging the passivation layer and promoting the Cu film’s corrosion behavior. Simultaneously, it forms an oxidation layer which acts as a passivation layer to protect the film. The possible reactions are shown in the below equations [48], [49]:

(2) Cu + Cl^−^ → CuCl + e^−^

(3) CuCl → Cu^2+^ + Cl^−^ + e^−^

(4) 2CuCl_2_ + H_2_O → Cu_2_O + Cl^−^ + H_2_↑

**Table 3:** Fitted EIS results of fabricated films.

Electrodeposition Methods	R_s_ (Ω)	R_p_ (kΩ)	CPE (μΩ^−1^ × s^n^)
Conventional	6.8	1.37	1058.75
SC-CO_2_	3.79	1.57	871.6
US-SC-CO_2_ @ 15 W cm^−2^	4.54	2.72	628.14
US-SC-CO_2_ @ 30 W cm^−2^	4.09	2.21	675.77

**Fig. 11:**
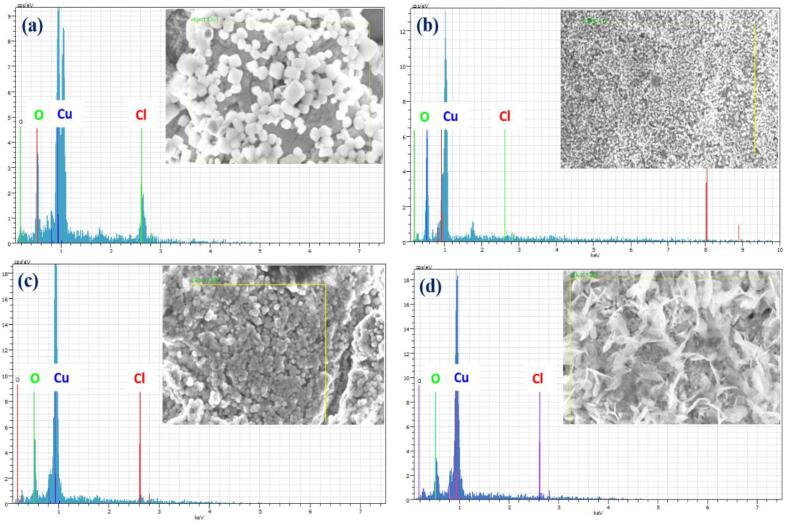
EDX analysis of (a) conventional, (b) SC-CO_2_, (c) US-SC-CO_2_ @ 15 W cm^−2^, (d) US-SC-CO_2_@ 30 W cm^−2^ after corrosion analysis.

Commonly, metal oxides are more robust than pure metals to protect the film’s surface from a corrosive medium. Therefore, R_ct_ values are increased due to the formation of oxide as a passivation layer that slows down the charge transfer rate. CPE value is related to the surface roughness, and it is considered the contact area with a NaCl solution [50]. To validate the possible reaction, films are examined using EDX analysis after corrosion, and the results are displayed in Fig. 11(a–d). The clear evidence of oxidized passive layers is confirmed with morphological changes and quantitative analysis. In the US-SC-CO_2_ (42 kHz @ 15 W cm^−2^) method, the metal ions influenced by the cavitation effect results in closer arrangement on the surface of the electrode. This result is verified with morphological studies. Therefore, the corrosive ions are not able to penetrate the compact surface easily. At the same time, the increased ultrasonic power (42 kHz/30 W cm^−2^) results in adverse effects due to the pinhole generation. In this case, the Cl^-^ ions easily penetrate through the pinhole and affect the surface area. Thus, US-SC-CO_2_ @ 42 kHz/30 W cm^−2^ is not enhancing the polarization resistance further. Hence, the optimum parameters of ultrasonic power could be suggested to the lower level of US-SC-CO_2_ @ 42 kHz/15 W cm^−2^.

## 4 Conclusions

In summary, the present study investigates the influence of ultrasonic power density on the electrodeposition of Cu films through the novel US-SC-CO_2_ electrodeposition process. The characteristics changes of the surface morphology and surface roughness are observed with FESEM and AFM techniques. At the optimized condition, the utilization of US-SC-CO_2_ has resulted in a fine morphology with smoother surface roughness. The ultrasound’s influence in the electrodeposition process is apparent with the decreased peak intensity and grain size. Corrosion studies application is also demonstrated with two analytical techniques: electrochemical impedance spectroscopy and linear polarization analysis. The corrosion studies revealed that the best polarization resistance is achieved by the US-SC-CO_2_ method. However, the increased power density leads to an adverse effect on corrosion resistance. Therefore, the application of US-SC-CO_2_ to electrodeposition methods would benefit from a low ultrasound power density of 15 W cm^−2^.

## CRediT authorship contribution statement

**Sabarison Pandiyarajan:** Conceptualization, Methodology, Software, Investigation, Writing - original draft. **Po-Ju Hsiao:** Conceptualization, Methodology, Software, Investigation, Writing - original draft. **Ai-Ho Liao:** Resources, Supervision, Writing - review & editing. **Muthusankar Ganesan:** Data curation, Formal analysis, Visualization, Writing - review & editing. **Shobana Sebstin Mary Manickaraj:** Data curation, Formal analysis, Visualization, Writing - review & editing. **Chen-Ta Lee:** Data curation, Formal analysis, Visualization, Writing - review & editing. **Sheng-Tung Huang:** Resources, Supervision, Writing - review & editing. **Ho-Chiao Chuang:** Resources, Supervision, Writing - review & editing.

## Declaration of Competing Interest

The authors declare that they have no known competing financial interests or personal relationships that could have appeared to influence the work reported in this paper.
